# Assessment of Feasibility of Face Covering in School-Aged Children With Autism Spectrum Disorders and Attention-Deficit/Hyperactivity Disorder

**DOI:** 10.1001/jamanetworkopen.2021.10281

**Published:** 2021-05-17

**Authors:** Benjamin Aaronson, Sara Nelson Glick, Christa Jefferis Kirk, William A. McCloud, Tyler R. Sasser, Danielle M. Zerr, Janet A. Englund

**Affiliations:** 1 Department of Pediatrics, University of Washington, Seattle; 2 Division of Allergy and Infectious Diseases, Department of Medicine, University of Washington, Seattle; 3 Department of Pharmacy, Seattle Children’s Hospital, Seattle, Washington; 4 University of Washington Autism Center, Seattle; 5 Department of Psychiatry and Behavioral Sciences, Seattle Children’s Hospital, Seattle, Washington; 6 Division of Infectious Disease, Department of Pediatrics, Seattle Children’s Hospital, Seattle, Washington

## Abstract

This cohort study reports the outcomes of using positive behavior supports to promote masking in school-aged children with autism spectrum disorders (ASD) and/or attention-deficit/hyperactivity disorder (ADHD) attending a summer day treatment program.

## Introduction

The effect of pediatric transmission of SARS-CoV-2 on the spread of COVID-19 remains a concern in creating policies for school reopening.^1,2^ Although children have a lower prevalence of developing overt COVID-19 symptoms than adults, asymptomatic transmission may contribute to the spread of COVID-19.^3^ Cloth face coverings are considered a key strategy in reducing the spread of SARS-CoV-2. However, policy makers and parents are concerned about students’ ability to mask effectively.^4^ We report the outcome of using positive behavior supports to promote masking in verbal school-aged children with autism spectrum disorders (ASD) and/or attention-deficit/hyperactivity disorder (ADHD) attending a summer day treatment program.

## Methods

In this cohort study, we examined face covering behavior in 104 children aged 5 to 13 years who participated in a summer treatment program at the University of Washington (UW) Autism Center during July 2020. Informed consent was obtained verbally, and all procedures were reviewed and approved by the UW institutional review board. This study followed the Strengthening the Reporting of Observational Studies in Epidemiology (STROBE) reporting guideline. Children attended the 4-week day camp on weekdays for 6 hours/day. Groups of 10 children were supervised by 6 to 7 graduate and undergraduate students. Activities included group discussions, sports, board games, snack and lunch, and recess; all activities were held outdoors. A token economy allowed children to earn points for behaviors, including helping, sharing, following activity rules, and sportsmanship. Activities were divided into 10- to 15-minute intervals,^5^ and behavioral data were taken continuously. A masking bonus was recorded by a research assistant for each interval during which a child properly wore a face covering over their nose and mouth for most of the interval. Points were redeemed at the end of the day for small prizes. We analyzed data on face covering during the second, third, and fourth weeks of the program Monday through Thursday (Fridays followed a different schedule). Data from the first week were excluded, as the research protocol and tracking were under development. Demographic data and clinical diagnoses were reported by guardians at enrollment as part of intake paperwork. The association between frequency of face covering and a child’s age was assessed using simple linear regression (α = .05; 2-tailed) in Stata SE version 16.1 (StataCorp).

## Results

**Figure 1.**
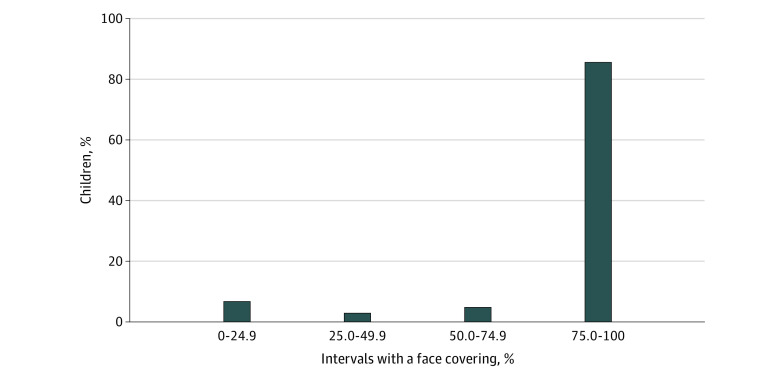
Proportion of Children Observed Wearing a Face Covering Across Observed Intervals During the Summer Treatment Program

**Figure 2.**
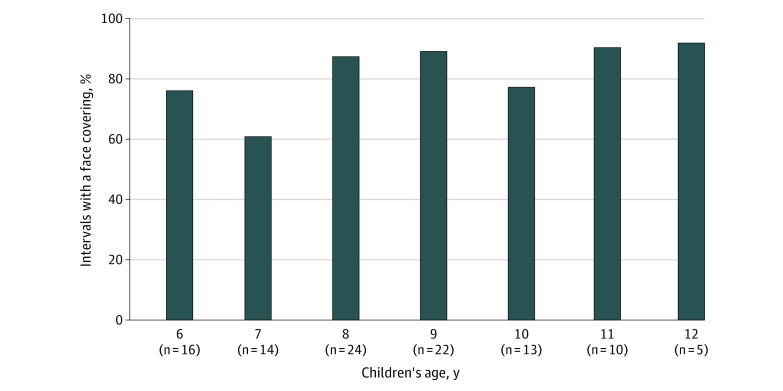
Proportion of Intervals Children Were Observed Wearing a Face Covering During the Summer Treatment Program by Age

Our sample included 104 children with a mean (SD) age of 8.9 (1.7) years; 84 (81%) reported a gender of male, 89 (86%) attended public school, and 71 (68%) had an individualized education program. Reported race/ethnicity included 1 child (1%) who reported being American Indian or Alaska Native; 17 (16%), Asian; 4 (4%), Black or African American; 5 (5%), Hispanic or Latino; 71 (68%), White; and 16 (16%), other or multiple categories. Clinical diagnoses were as follows: ASD, 28 (27%); ADHD, 37 (36%); ASD and ADHD, 29 (28%); and none, 11 (10%). During the 3-week period, staff recorded face coverings during 30 539 total intervals (range, 175-331 per child) with an mean (SD) of 24.47 (4.85) intervals per day per child. During this period, 89 children (86%) wore a face covering during at least 75% of all observed intervals (Figure 1). This included intervals for snack and lunch, when face coverings were not expected. There was a significant association between age and the prevalence of wearing a face covering, with face coverings observed during 2682 of 4411 intervals (61%) among children aged 7 years and 1147 of 1248 intervals (92%) among children aged 12 years (*P* = .01) (Figure 2).

## Discussion

Our carefully tracked interval data indicate that a group of school-aged children, most of whom had ASD and/or ADHD, were capable of face covering across activities. Our findings are consistent with recent reports of face covering in school-aged children^6^ but included direct observation and extended to children with greater special education needs. Limitations include a small sample size, a high staff to child ratio, no data on prior masking behavior, and no interrater reliability. As policy makers and school personnel consider plans for in-person activities, face covering can be used as part of a constellation of practices to reduce the transmission of SARS-CoV-2 in pediatric settings.
